# Molecular Identification of a Moricin Family Antimicrobial Peptide (Px-Mor) From *Plutella xylostella* With Activities Against the Opportunistic Human Pathogen *Aureobasidium pullulans*

**DOI:** 10.3389/fmicb.2019.02211

**Published:** 2019-10-11

**Authors:** Xiaoxia Xu, Anqiao Zhong, Yansheng Wang, Boda Lin, Peng Li, Wenyan Ju, Xiaojia Zhu, Jing Yu, Surajit De Mandal, Fengliang Jin

**Affiliations:** ^1^Department of Entomology, Key Laboratory of Bio-Pesticide Innovation and Application of Guangdong Province, College of Agriculture, South China Agricultural University, Guangzhou, China; ^2^Department of Respiratory Medicine, Yidu Central Hospital, Weifang, China; ^3^State Key Laboratory of Respiratory Disease, Guangzhou Institute of Respiratory Disease, The First Affiliated Hospital of Guangzhou Medical College, Guangzhou, China

**Keywords:** antimicrobial peptides, moricins, qRT-PCR, antifungal agent, *Plutella xylostella*, *Aureobasidium pullulan*

## Abstract

Antimicrobial peptides (AMPs) represent the largest group of endogenous compounds and serves as a novel alternative to traditional antibiotics for the treatment of pathogenic microorganisms. Moricin is an important α-helical AMP plays a crucial role in insect humoral defense reactions. The present study was designed to identify and characterize novel AMP moricin (Px-Mor) from diamondback moth (*Plutella xylostella*) and tested its activity against bacterial and fungal infection including the opportunistic human pathogen *Aureobasidium pullulans*. Molecular cloning of Px-Mor using rapid amplification of cDNA ends revealed a 482 bp full length cDNA with 198 bp coding region. The deduced protein sequence contained 65 amino acids, and the mature peptides contained 42 amino acid residues with a molecular mass of 4.393 kDa. Expression analysis revealed that Px-Mor was expressed throughout the life cycle of *P. xylostella* with the highest level detectable in the fourth instar and prepupa stage. Tissue specific distribution showed that Px-Mor was highly expressed in fat body and hemocyte. *In vitro*, antimicrobial assays indicated that Px-Mor exhibited a broad antimicrobial spectrum against Gram positive bacteria (GPB), Gram negative bacteria (GNB) and fungi. Moreover, scanning electron microscopy and transmission electron microscopy (TEM) revealed that Px-Mor can cause obvious morphological alterations in *A. pullulans*, which demonstrated its powerful effect on the mycelia growth inhibition. Taken together, these results indicate that Px-Mor plays an important role in the immune responses of *P. xylostella* and can be further exploited as an antimicrobial agent against various diseases including for the treatment of *A. pullulans* infection.

## Introduction

Insect-pathogen association can be seen as an evolutionary arms race where pathogen evolves to optimize host infection and its dispersion; in turn, the host (insect) activates immune response involving humoral and cellular reaction to defense against the infection. Being devoid of acquired immunity, insects developed efficient innate immune system (IIS) (Hoffmann and Reichhart, 2002) which rely on various mechanisms: such as phagocytosis, activation of proteolytic cascades including coagulation and melanization as well as production of different antimicrobial peptides (AMPs) (Richman and Kafatos, 1996; Boman, 1998; Boulanger et al., 2001). AMPs involved in insect humoral defense reactions and were rapidly secreted into the hemolymph upon microbial infection (Kanost et al., 2004). These AMPs can serve as novel alternatives to traditional antibiotics for the treatment of pathogenic microorganisms due to their broad spectrum of activity, less potential for resistance development, and capability to modulate the host immune response (Mohammad et al., 2015; Mohamed et al., 2016).

To date, a large number of insect AMPs have been identified and classified into four groups according to their basic structure, including the linear α-helical (cecropin and moricin), glycine-rich (attacin and gloverin), proline-rich (drosocin, and lebocin), and cysteine-rich peptides (insect defensin and drosomycin) (Boman, 2003; Bulet and Stocklin, 2005; Yi et al., 2014). A broad range of AMPs including cecropins, moricins, gloverins, attacins, lebocins, and defensins have been reported in Lepidopteran insects (Ponnuvel and Yamakawa, 2002; Cheng et al., 2006). Moricins are important α-helical cationic AMPs which play an important role in insect humoral defense reactions. This type of peptide was initially isolated from the hemolymph of *E. coli* immunized *Bombyx mori* larvae (Bm-moricin A1) and contains 42-amino-acid peptide (Hara and Yamakawa, 1995). Later on, Bm-moricin2 and other moricin-like variants were also reported in the *B. mori* genome (Xia et al., 2004; Cheng et al., 2006). Recently, several moricin genes were identified in *Galleria mellonella* (Brown et al., 2008) and *Manduca sexta* (Zhu et al., 2003), and another moricin analogues peptide was isolated from *Spodoptera litura* (Oizumi et al., 2005) and *Helicoverpa armigera* (Wang et al., 2010). Previous studies reported that the AMP moricin has a high activity against Gram-positive and Gram-negative but is less active against yeasts. Moricin has been shown to be membrane active as it affects the permeability of cytoplasmic membranes (Hara and Yamakawa, 1995). In addition, an artificial moricin gene was synthesized and expressed in *Escherichia coli* (Hara and Yamakawa, 1996; Shen et al., 2010), and it was confirmed that the physical and biological characteristics of the recombinant moricin were identical with their natural counterparts. The recombinant moricin efficiently suppressed the growth of methicillin-resistant *Staphylococcus aureus* (Hara and Yamakawa, 1996). But in-depth studies of the interaction between moricin with the fungal pathogen are not yet performed.

The black yeast-like fungi *Aureobasidium pullulans*, can produce yeast-like hyaline conidia as well as a filamentous growth form (Taylor et al., 2006). It is also known as an important producer of the biodegradable extracellular polysaccharide (EPS) pullulan (Zalar et al., 2008). Despite the biotechnological significance, *A. pullulans* is considered as an opportunistic human pathogen and causes illness in immunocompromised patients (Chan et al., 2011). It also causes a variety of localization as well as rare systemic infections in humans (Hawkes et al., 2005) including diseases such as catheter-related septicemia (Huang et al., 2008), fungemia, nosocomial infection (Bolignano and Criseo, 2003), abscess in the spleen, peritonitis (Clark et al., 1995), pneumonia, meningitis, corneal ulcer (Jones and Christensen, 1974), invasive pulmonary infection, scleral infection (Panda et al., 2006), cutaneous infection (Joshi et al., 2010) and asthma (Niedoszytko et al., 2007). Its increased indoor concentrations have also been correlated to various health symptoms (Su et al., 1992). Therefore, a potential antimicrobial agent is needed to control this opportunistic human pathogen.

In the present study, we report the characterization of a new antibacterial peptide moricin (Px-Mor) from the lepidopteran insect, *P. xylostella*. Furthermore, we investigated the antimicrobial activity and mechanism of the purified recombinant Px-Mor against the mycelia of the opportunistic human pathogen *A. pullulans*.

## Materials and Methods

### Insects and Microorganisms

The larvae of *P. xylostella* were collected from the vegetable fields of South China Agricultural University. The insects were reared on cabbage mustard at 25 ± 2°C, 60–70% RH for a 16:8 h light/dark photoperiod. Microorganisms including Gram-positive bacteria (*Staphylococcus aureus*, *Bacillus cereus*, and *B. subtilis*); Gram-negative bacteria (*E. coli DH5α*, *E. coli K12D31*, *Pseudomonas fluorescent*, and *Salmonella choleraesuis*); and fungi (*A. pullulans*, *Aspergillus flavus*, *Botrytis cinerea*, *Peronophythora litchi*, *Aspergillus fumigatus*, and *Fusarium oxysporum*) were obtained from the Research Institute of Microbiology, Guangzhou, China. *E. coli* DH5a, BL21 (DE3), and *B. thuringiensis* were grown in LB broth at 37°C until they reached the mid-log (2–7 × 10^5^CFU/ml) phage. Whereas *A. pullulans*, was grown on potato dextrose agar (PDA) plates and incubated at 26 ± 2°C for 10 days.

### RNA Extraction and Reverse Transcription

Total RNA was extracted from the composite sample of four larvae (each instar), four pupae and four adults of *P. xylostella* using Trizol reagent following the manufacturer’s protocol. First-strand cDNA was synthesized by oligo-dT_18_ primer and Super-script III Reverse Transcriptase (TaKaRa, Japan) in reaction volume of 20 μl. The reaction was incubated at 42°C for 60 min, and then was maintained at 70°C for 15 min. The resultant cDNA was used as the template for ORF sequence amplification. The total RNA, which was extracted from the 4^th^ instar larvae of *P. xylostella* after immunization with heat-inactivated *B. thuringiensis*, was used to synthesize 5′- and 3′-cDNA. The 5′- and 3′-cDNA were obtained by using SMARTer RACE 5′/3′Kit (Clontech, USA) according to the manufacturer’s protocol. Prior to RNA extraction from different tissues, hemocyte, fat body, Malpighian tubule and midgut were isolated from the 4^th^ instar larvae which were induced by PBS, and *E. coil*, heat-inactivated and active *B. thuringiensis*. The synthetic cDNA of these samples was served as a template for real time PCR.

### Cloning the Full-Length cDNA of Px-Mor

Px-Mor unigene sequences, which were obtained from *P. xylostella* transcriptome, were verified through RT-PCR. PCR was performed with forward primer Moricin F:5′-CTTCCACTTGCTGATGCTGGCGC-3′ and reverse primer Moricin R:5′-CTGTTCCGCACGTGGTTGTACAC-3′ by the following reactions: 94°C 5 min; 94°C, 30 s, 59°C, 30 s, 72°C, 30 s, 30 cycles; 72°C, 8 min. Amplified PCR products were detected on a standard 1.0% (w/v) agarose gel and purified by E.Z.N.A.™ Gel Extraction kit (OMEGA, USA). The purified PCR product was cloned into pMD18-T vector (TaKaRa, Japan) as per the manufacturer’s instructions. Both 5′- and 3′ Rapid Amplification of cDNA Ends reactions were primed with the gene specific primer and the universal primer mix (UPM) supplied in SMARTer RACE 5′/3′Kit (Clontech, USA). The nested gene specific primers, which contained two 5′-GSP and two 3′-GSP, were designed based on the verified unigene sequences of Moricin. In 5′-RACE reaction, the two 5′-GSP, which consisted of 5′-Moricin R1: 5′-GCGTTGACGTTGACCTTGGGCGCGG-3′ and 5′-Moricin R2: 5′-GCAGCGTCATGGCCGCCAGCGCCAGC-3′, were the reverse primers, while the UPM was the forward primer. In 3′-RACE, the two 3′-GSP, which contained 3′-Moricin F1:5′-CGCGCCCAAGGTCAACGTCAACGCC-3′ and 3′-Moricin F2: 5′-GGGACG GCGCATGAAGTGTACAACC-3′, were the forward primers. The RACE-PCR was carried out by the touchdown PCR program as follows: after denaturation for 2 min at 94°C, 5 cycles at 94°C for 30 s, 70°C for 30 s and 72°C for 1 min were used, continuing with 30 cycles of 94°C for 30 s, 68°C for 30 s and 72°C for 1 min, and finally 72°C for 8 min. Nested PCR reactions were performed using the 50 times diluted product of the first PCR under the conditions described above with each gene specific primers and NUP (UPM short) supplied in the kit. Generated fragments of the 5′- and 3′-RACE-PCR were cloned into pMD18-T vector (TaKaRa, Japan).

### Sequences Analysis of Moricin

The obtained cDNA sequence were blasted to NCBI through the Internet to estimate the degree of homology to known antibacterial peptides. Other reported Lepidoptera Moricin sequences were retrieved from the GenBank database^1^ for phylogenetic analysis. The signal peptide was analyzed by SignalP^2^ and the theoretical isoelectric point (pI) was calculated by the compute pI software^3^. The predicted molecular weight, and charges of the protein at pH 7.00 were estimated using Protein Calculator v3.3^4^. The sequences were aligned in Clustal X. 2.0^5^. The phylogenetic tree was generated using the neighbor-joining method in MEGA 6.0 (Tamura et al., 2013). The secondary and tertiary structure was predicted by the homology modeling method on Phyre 2^6^.

### RT-qPCR of Px-Mor

Assessment of the mRNA expression level of Px-Mor during the developmental stages in 4^th^ instar *P. xylostella* larvae was carried out after the induction of inactivated *E. coil*, *B. thuringiensis*, *A. pullulans*, and PBS (as bacteria control), Tween-80 (as fungi control). Fat body and hemocytes were isolated from the inactivate pathogen induced *P. xylostella* larvae after 0, 6, 12, 18, 24, 30, 36, 42, and 48 h followed by wash three times using 1 × PBS buffer. The primers for RT-qPCR amplification (F: 5′-TTTGATGCTGGCTCTGGT GG-3′ R: 5′-GCCCTTCTTGAGTGCGTTGA-3′) were designed according to ORF of moricin genes. The final reaction mixture contained 1 μl of each primer, 12.5 μl of SYBR® Green PCR Master Mix, and 2 μl cDNA. The reaction were subjected to: 95°C for 3 min followed by 39 cycles at 95°C for 10 s, 58°C for 30 s, and ending with 95°C for 10 s. The partial fragment of the muscle-actin gene (NCBI accession no. AB282645) was served as an internal control and amplified from the same cDNAs with the Actin qF/qR primers. Melting curve analysis was applied to all reactions to ensure homogeneity of each reaction product. Three biological replications (*n* = 3) were performed for the each reaction and the2^−ΔΔCt^ method was used to measure the relative transcription levels.

### Construction of Expression Vectors pMTHisAPx-Mor

The cDNA sequence encoding mature Px-Mor was amplified by PCR using primers, i.e., Px-Mor F:CCG***AGATCT***ATGAGATTCTTGCACTTGCTGATGCTGGCG and Px-Mor R:GCC***ACCGGT***CCCCTGGTTCCTGTTCCGCACGT. The purified PCR fragments were digested with Bgl II and AgeI enzymes and ligated into the Bgl II /AgeI-digested expression vector pMT/BiP/V5-HisA (Invitrogen, USA) and transformed into the competent cells DH5α. The recombinant expression plasmid pMTV5HisAPx-Mor was confirmed by DNA sequencing. The positive plasmid was transient transfected into *Drosophila Schneider* S2 and the expression was further confirmed by SDS-PAGE and western blot analysis. For transfection assay, *Drosophila Schneider* S2 cells were maintained at 28°C in incubator and seeded overnight in serum-free medium, and Lipofectamine 2000 (Invitrogen, USA) was used for transfection based on the protocol. In order to generate stable S2 cells expressing Px-Mor, the recombinant expression vectors pMTHisAPx-Mor was transfected into S2 (5 × 10^6^ cells/ml). The pCoBlast plasmid was used for stable selection. Calcium Phosphate Transfection kit (Invitrogen, USA) was used for the transfection of the S2 cells. The cells were further transferred into a selection medium containing 10 μg/ml blasticidin. The blasticidin-resistant clones appeared in about 2 weeks and were further expanded in selection medium to express the Px-Mor in S2 cells with the induction of 500 μM copper sulfate. These cultures were harvested after 7 days cultivation. The media and cell lysate were obtained by centrifugation at 800 rpm for 3 min. And the expression protein was analyzed by SDS-PAGE and western blot.

### Expression and Purification of Recombinant Protein Px-Mor

Large scale expression of Px-Mor were carried out by expanding the stably transfected S2 cells in large suspension cultures. In 150-cm^2^ flasks, cell culture medium was collected every 24 h after copper sulfate (final concentration 500 μM) induction. The cell culture medium was collected and combined 10 days collection, and centrifuged with 3,000 rpm 10 min at 4°C, the debris was removed and cell free medium was collected. This cell free medium was then thoroughly dialyzed multiple times using an 3 kDa molecular weight cut off, membrane tubing into a buffer containing 20 mM Tris pH 8.0, 500 mM NaCl, and 1% glycerol to completely remove the copper ions. About 1 ml His Mag TM Agarose Bead (Novagen, USA) was used to purify the protein. Fractions were analyzed by Tricine-SDS-PAGE. All the fractions containing Px-Mor were collected, concentrated with PEG2000, dialyzed in ddH_2_O.

### Synthesis of Px-Mors and Melittin

According to the mature peptides amino acids, Px-Mor, Px-Mor113/114, Px-Mor115, and melittin were synthesized using the solid phase method and standard 9-fluorenyl methoxy carbonyl chemistry and purified to >95% purity using reverse-phase high-pressure liquid chromatography at the Peptide Synthesis Core Facility of the Qiangyao Biotechnology Company (Shanghai, China). The C-terminus was amidated to prevent degradation and improve the activity of the proteins.

### Antimicrobial Activity Assays

The antimicrobial activity of the purified recombinant Px-Mor was tested against several GPB, GNB, and fungi. The MICs was determined with the reference to the Clinical and Laboratory Standards Institute guidelines (Wikler, 2009). The minimal growth inhibition concentration (Tavares et al., 2012) was analyzed using a liquid growth inhibition assay and expressed as the lowest concentration of the peptide at which no growth was found, while the activity was determined by monitoring the absorbance at 494 nm.

### Hemolytic Assay

The hemolytic activity of the recombinant protein Px-Mor and synthesized Px-Mor, Px-Mor113/114, Px-Mor115, and Melittin were performed using human red blood cells (RBCs) isolated from heparinized blood (Jin et al., 2006). The HBCs were prepared from freshly collected human blood (4 ml) by centrifugation at 1,500 rpm for 10 min at 4°C. The cells were washed three times with 0.01MPBS (NaCl 0.8 g/L, KCl 0.2 g/L, KH_2_PO_4_ 0.2 g/L, and Na_2_HPO_4_ 1.15 g/L, pH 7.2) and suspended as 10% suspension in hematocrite solution. The human HBCs were incubated with dissolved in PBS protein (purified recombinant protein Px-Mor and synthesized Px-Mor, Px-Mor113/114, Px Mor115, and Melittin) for 1 h at 37°C. The concentrations of tested proteins were: 100, 50, 25, 12.5, 6.25, 3.125, 1.56, and 0.78 μM. The samples were centrifuged at 3,500 rpm for 5 min and the absorbance of the supernatant was measured at 414 nm with an ELISA plate reader. Zero hemolysis and 100% hemolysis were determined in PBS and Triton X-100, respectively.

### Scanning Electron Microscopy and Transmission Electron Microscopy

The electron microscopy was used to investigate recombinant moricin interacting with *A. pullulans* mycelia (Niedoszytko et al., 2007). The mycelia were incubated with 30 μM recombinant proteins moricin at 28°C for 5 days, and then collected (0.5 cm × 0.5 cm) followed by a twice wash with 0.1 M phosphate buffer. The suspension were fixed in 2.5% glutaraldehyde in 0.1 M phosphate buffer for 3 h, and washed three times with 0.1 M phosphate buffer followed by dehydration in ascending series of ethanol (50, 70, 80, 90, and 100%, 15 min each). Samples were dried for few seconds in room temperature and mounted on the SEM stubs using double-sided carbon tape. Dried samples were sputtered with the gold and analyzed with the SEM under Quanta 200 FEG at high-vacuum mode. For TEM analysis *A. pullulans* spores (1 × 10^6^ conidia/ml) were treated with 30 μM Px-Mor at 37°C for 30 min. Finally the supernatant was collected using centrifugation (5,000 *g* for 5 min) and cold glutaraldehyde (0.5%, w/v, in 0.1 M sodium cacodylate buffer, pH 7.4) was added and incubated for 2 h at 4°C.

### Data Analysis

Figures were made with the GraphPad Prism 7.0. All the statistical analysis was conducted using the SPSS software; data were analyzed using Student’s *t*-test for significant differences between the treatment groups and the control. One-way analysis of variation (ANOVA) followed by Tukey’s multiple comparisons was used to compare the relative expression of Px-Mor transcript in different development stages or tissues.

## Results

### Sequence Characteristics of Full-Length Px-Mor

**Figure 1:**
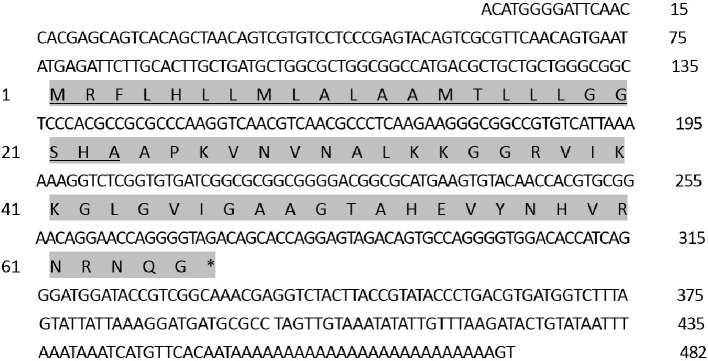
Nucleotide and deduced amino acid sequences of *Px-Mor* cDNAs from the diamondback moth, *Plutella xylostella* (GenBank: No.KF960047). The stop codons are indicated by an asterisk. Putative signal peptides at the N-termini are underlined.

The RT-PCR approach performed with a pair of primers designed from *P. xylostella* transcriptome identified the cDNA fragment (Moricin). By RACE-PCR strategies, a full-length cDNA, encoding antimicrobial peptide gene Moricin, was cloned from *P. xylostella*. The nucleotide and deduced amino acid sequences are shown in Figure 1. The full-length cDNA of Moricin was 482 bp. The open-reading frame (ORF) is 198 bp in length and encodes a precursor with 65 amino acid residues, which we have named Px-Mor. The name was assigned according to the high identity of this protein with proteins from lepidopteran insects in BLAST searches. The sequence for Px-Mor have been deposited in GenBank under the accession number KF960047. The deduced amino acid sequence for moricin possesses a putative signal sequence of 23 amino acid residues. The predicted mature Px-Mor consists of 42 amino acid residues with a molecular mass of 4.393 kDa and a pI of 11.17. The secondary structure comprised α-helix and turns and its tertiary structure are shown in Supplementary Figure S1.

**Figure 2:**
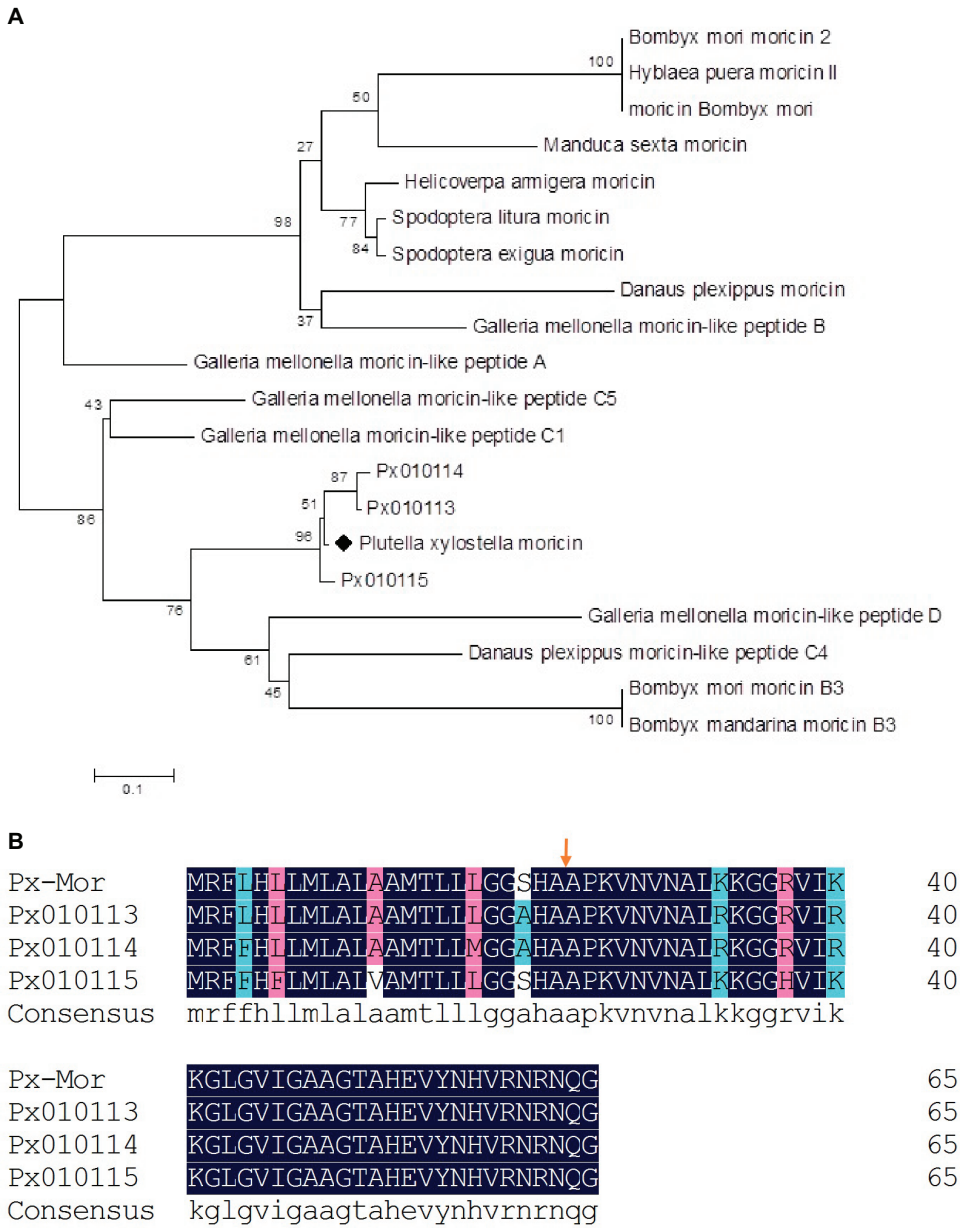
**(A)** Phylogenetic analysis of the lepidopteran moricin family. Computer-aimed phylogenetic analysis was done by the neighbor-joining method for amino acid sequences among moricin described above. A phylogenetic tree was constructed using Clustal X. Genbank accession numbers of the amino acid sequences used for the comparison are BAC79440 (*S. litura* moricin), AAT3887 (*S. exigua* moricin), BAB13508 (*B. mori* moricin), ADR51149 (*H. armigera* moricin), EHJ70108 (*D. plexippus* moricin), BAA77338 (*B. mori* moricin 2), AAW21269 (*H. puera* moricin II), AAO74637 (*M. sexta* moricin), AEM66445 (*B. mori* moricin B3), AEM66435 (*B. mandarina* moricin B3), ABQ42579 (*G. mellonella* moricin-like peptide C5), EHJ73174 (*D. plexippus* moricin-like peptide C4), ABQ42574 (*G. mellonella* moricin-like peptide B), ABQ42580 (*G. mellonella* moricin-like peptide D), ABQ42575 (*G. mellonella* moricin-like peptide C1), and ABQ42573 (*G. mellonella* moricin-like peptide A). Px010113, Px010114, and Px010115 come from *P. xylostella* genome. **(B)** Comparison of the predicted amino acid sequences of moricins from *P. xylostella*. The alignment of *Px-Mor*, Px010115, Px010114, and Px010113 was conducted using the DNAMAN. Px010115, Px010114, and Px010113 is the amino acid sequence of moricin from *P. xylostella* genome. The same amino acid is remarked with blue box. Before the arrows is the putative signal sequence of 23 amino acid residues and after the arrows is the mature peptide.

We aligned the amino acid sequence of Px-Mor with moricins from *B. mori* and 14 other species of Lepidoptera. Px-Mor displayed high identities with moricin-like peptide A from *G. mellonella* and moricin-like peptide C4 from *Danaus plexippus*, sharing 64% identity, and 63% identity, respectively. A phylogenetic tree was constructed by neighbor joining method in Figure 2, which indicated homology of Px-Mor with several Lepidopteran’s moricins. Px-Mor showed maximum homology with moricins from other lepidopteran insects, and was more close to *G. mellonella*, *D. plexippus*, and *B. mori*, which suggested that they might possess similar functions (Figure 2). We also aligned the amino acid sequence of Px-Mor with those predicted from the genome of *P. xylostella*. These uncharacterized proteins, which were referred to as Px010113, Px010114, and Px010115, showed 92.21 to 95.38 (based on the premature peptide) or 95.24 to 97.62 (based on the mature peptides) of the identities to Px-Mor (Figure 2B). The secondary structure and peptide characteristics of the Px-Mor analogues are shown in Supplementary Table S1.

### Px-Mor Expression Patterns in *P. xylostella*

**Figure 3:**
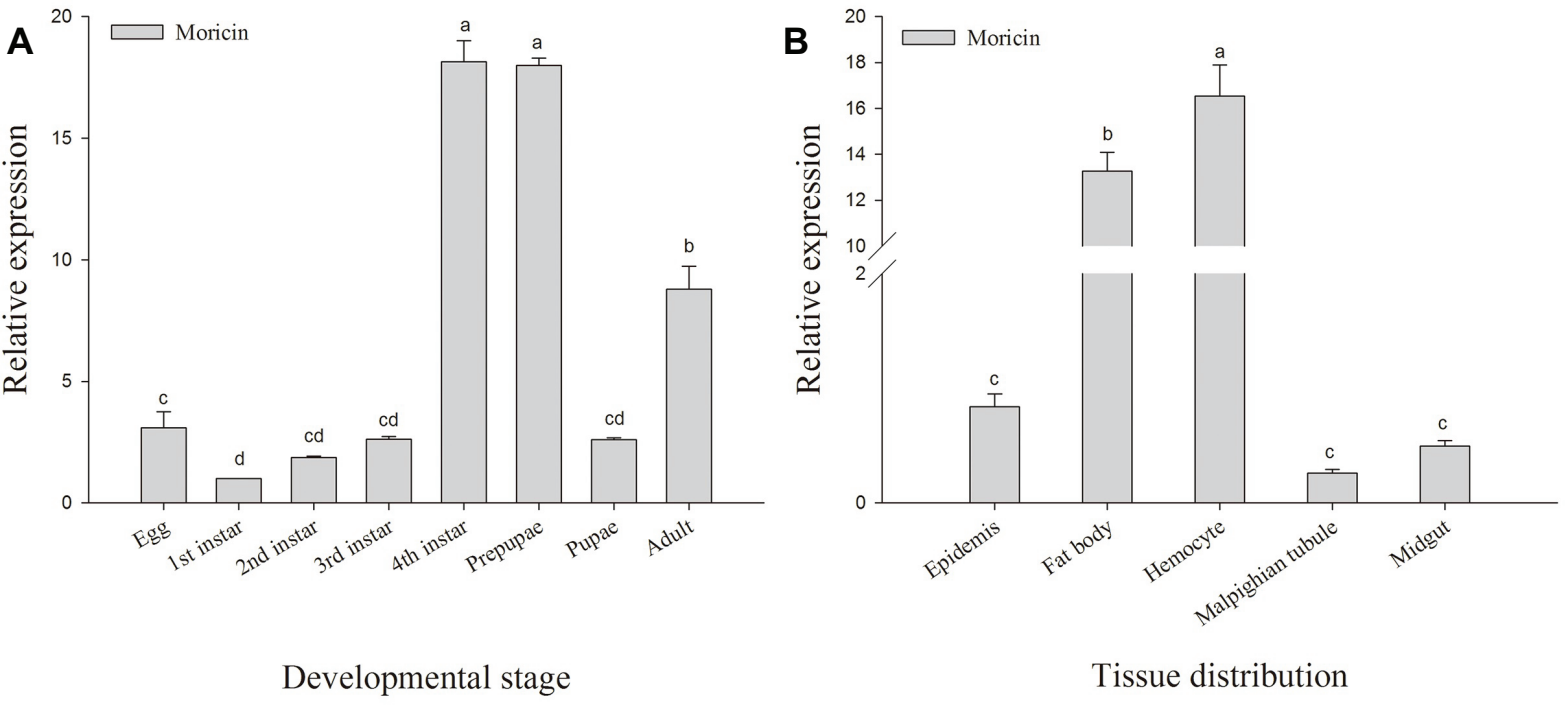
Spatial and temporal expression difference *Px-Mor* in *P. xylostella*. **(A)** Developmental expression patterns of *Px-Mor* in *P. xylostella* from egg to adult. **(B)** Tissue distribution of *Px-Mor* in the fourth instar larvae of *P. xylostella*. The temporal and spatial expression of *Px-Mor* transcript levels in *P. xylostella* as detected by SYBR green qRT-PCR. Comparison of the level of *Px-Mor* mRNA (relative to β-actin mRNA) was performed by *t* test. Data with different letters are significantly different (*p* < 0.05) (DMRT) among different developmental stages and tissue distribution.

To investigate the transcript levels of *Px-Mor* in different developmental stages of *P. xylostella*, a real-time PCR was performed with different cDNAs from egg, larva, prepupa, pupa and adult stages as the template. *Px-Mor* was expressed throughout the life cycle of *P. xylostella* (Figure 3A). The relative expression levels of *Px-Mor* were highest in fourth instar and prepupa stage, suggesting that moricin played different roles at different developmental stages. The main tissue of *P. xyostella* related to immune response, including hemocyte, midgut, fat body, Malpighian tubule and epidermis, were isolated from the fourth instar larvae of *P. xyostella* to synthesize cDNA templates. To determine the tissue distribution of moricin at mRNA level, RT-qPCR experiments were performed. Moricin was detected in all of the tissues, mainly expressed in hemocyte and fat body (Figure 3B).

### The Relative Expression of Px-Mor Upon Infection With Different Microorganisms

**Figure 4:**
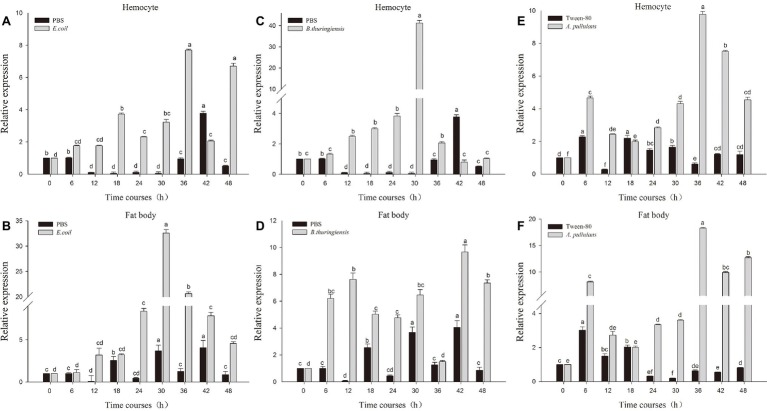
Relative quantification of expression of moricin genes from hemocytes and fat body after injection with different microorganisms. Actin was used as an internal control; PBS and Tween-80 were used as tests control. **(A,B)** The relative expression of moricin after injection with *E. coli* in the hemocyte and fat body of *P. xylostella*. **(C,D)** The relative expression of Px-Mor after injection *B. thuringiensis* in the hemocyte and fat body in *P. xylostella*, respectively. **(E,F)**. The relative expression of moricin after injection with *A. pullulans* in the hemocyte and fat body of *P. xylostella*. Three biological replications (*n* = 3) were conducted, and the 2^−ΔΔCt^ method was used to measure the relative transcription levels. Means with different number letters are significantly different (*p* < 0.05) (Duncan’s Multiple Range Test) among different time after treated with *E. coli*, *B. thuringiensis*, and *A. pullulans*.

The fourth instar larvae of *P. xyostella* were infected with heat-inactivated pathogen by microinjection technique. To determine whether heat-inactivated pathogen infection induced an immune response, we respectively extracted RNA of fatbody and hemocyte from larvae 0, 6, 12, 18, 24, 30, 36, 42, and 48 h post infection and probed the expression level of *Px-Mor* with specific primer by RT-qPCR. As shown in Figure 4, the expression level of *Px-Mor* was differently up-regulated after induction of microorganism in the tissues. The greatest up regulation was detectable in hemocyte and fat body. *Px-Mor* was highly expressed at the time point of 6 h after the infection with heat-inactivated *B. thuringiensis* in fat body (Figure 4D). In hemocyte, 30 h after *E. coli* infection, the expression level of *Px-Mor* gene rapidly increased to the maximum, which was 23-fold higher than that in the control group (Figure 4C). The bacteria *B. thuringiensis* can induce a strong expression of moricin in *P. xyostella*, rat two time points in fatbody, one after 12 h of infection and the other after 30 h (Figure 4B). The fungi *A. pullulans* also induced a strong expression of moricin in *P. xyostella*, which reached a high peak after 6 h infection and reached the maximum after 36 h in both fatbody and hemocyte (Figures 4E,F). We suspected that *Px-Mor* is mainly expressed in changeable period of metamorphosis of prepupa development, which illustrated that Moricin have certain biological significance during the process of metamorphosis.

### Recombinant Expression and Purification of Px-Mor

**Figure 5:**
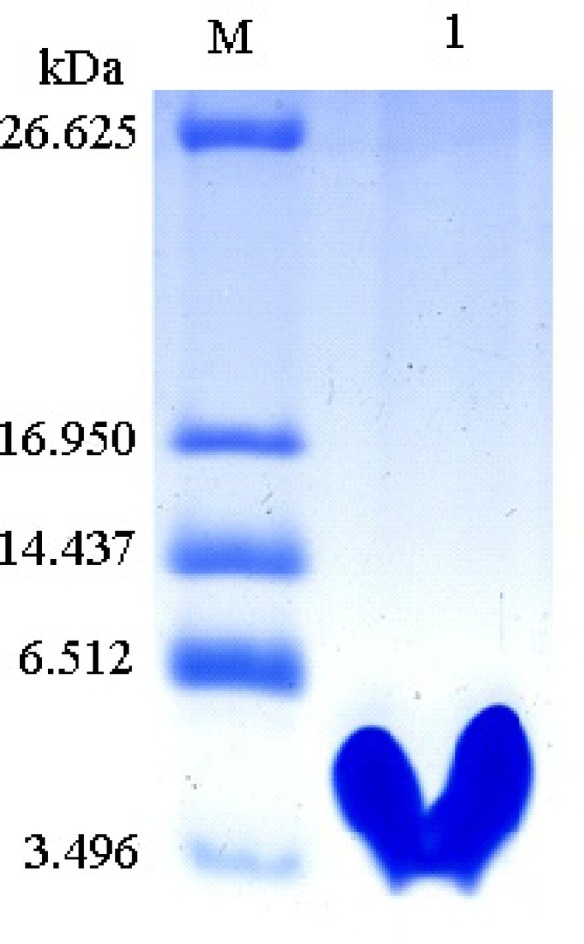
Tricine-SDS-PAGE analysis of the purified recombinant Px-Mor. Lane M, polypeptide SDS-PAGE molecular weight markers; Lane 1, 2 μg purified recombinant Px-Mor was analyzed using Tricine-SDS-PAGE. The proteins was stained with Coomassie brilliant blue.

Recombinant protein Px-Mor was produced as a fusion protein with a 6 × His tag. Stable S2 cells containing *Px-Mor* was expressed in S2 cells under the induction of 500 μM copper sulfate for a large scale, and the fusion peptide was purified to homogeneity using Ni^2+^-NTA column. The findings of the purified recombinant Px-Mor by Tricine-SDS-PAGE and western blot revealed a peptide of approximately 4.93 kDa which could strongly cross-react with anti-His antibody (Figure 5).

### Antimicrobial Spectrum of Px-Mors

Px-Mor peptide from *P. xyostella* was tested against Gram-positive, Gram-negative and fungi. The results clearly illustrate that the peptides exhibit a high activity against the tested microbes (Table 1). The most effective values were recorded from the purified recombinant Px-Mor against *Escherichia coli* K_12_D_31_ and *Escherichia coli* DH5α with MICs of 1.1 and 1.2 μM, respectively. Purified recombinant Px-Mor was also active against *A. pullulans* with the MIC value of 3.5 μM. Compared to the purified recombinant Px-Mor, the synthetic protein, Px-Mor, Px-Mor113/114, and Px-Mor 115 also exhibited a high activity against the tested target microorganisms. Px-Mor, Px-Mor113/114, and Px-Mor115 showed strong activities against *A. pullulans* with the MIC values of 3.6, 3.7, and 6.9 μM, respectively.

**Table 1:** Antimicrobial activity of AMPs against microorganisms.

Microorganisms	MIC (μM)			
	Recombinant Px-Mor	Px-Mor	Px-Mor113/114	Px-Mor115
**Gram-positive**				
*Staphylococcus aureus*	2.8	2.7	3.1	4.6
*Bacillus cereus*	4.5	4.5	3.7	5.7
*Bacillus subtilis*	6.3	6.4	6.7	8.2
**Gram-negative**				
*Escherichia coli* DH5α	1.2	1.2	1.3	2.1
*Escherichia coli* K12D31	1.1	1.0	1.4	1.9
*Pseudomonas fluorescent*	4.6	4.6	3.2	5.8
*Salmonella choleraesuis*	5.0	4.9	5.3	4.7
**Fungi**				
*Aureobasidium pullulans*	3.5	3.6	3.7	6.9
*Aspergillus flavus*	15	16	11	28
*Botrytis cinerea*	23	22	25	36
*Peronophythora litchi*	15.6	16.5	18.3	42
*Aspergillus fumigatus*	21	20	25	37
*Fusarium oxysporum*	8.9	8.8	9.8	24

*Data are collected as minimal inhibitory concentrations (MICs)a and expressed in μM*.

### Hemolytic Assays of Px-Mors

Hemolytic assay results showed that all the Px-Mor analogues investigated had no hemolytic activity against the human red blood cells (RBCs). It suggested that none of the Px-Mor analogues were observed to be toxic to the human RBCs, comparing with melittin, which lysed 100% of the human RBCs even at a low concentration of 0.78 μM (Table 2).

**Table 2:** Hemolytic activity of recombinant and synthesized Px-Mor peptides.

Peptides	Percentage hemolysis (μM)							
	100	50	25	12.5	6.25	3.125	1.56	0.78
Px-Mor-His (recombinant)	0	0	0	0	0	0	0	0
Px-Mor113/114 (synthesized)	0	0	0	0	0	0	0	0
Px-Mor115 (synthesized)	0	0	0	0	0	0	0	0
Px-Mor (synthesized)	0	0	0	0	0	0	0	0
Melittin (synthesized)	100	100	100	100	100	100	100	100

*The hemolytic activity of the peptides was evaluated by determining the hemoglobin release of 8% suspensions of fresh human erythrocytes at 414 nm*.

### Effect of Px-Moron *A. pullulans* Morphology

**Figure 6:**
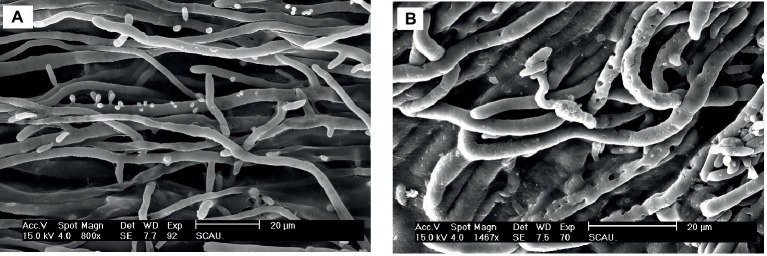
SEM analysis of the hypha of *A. pullulans* interacted with Px-Mor. **(A)** Naive *A. pullulans hypha*, **(B)** the hypha of *A. pullulans* interacted with Px-Mor. The SEM analysis showed that *A. pullulans hypha* became perforation and ruptured **(B)** after interacted with moricin from *P. xylostella* as compared to the untreated hypha **(A)**, which had a bright and normal smooth surface.

**Figure 7:**
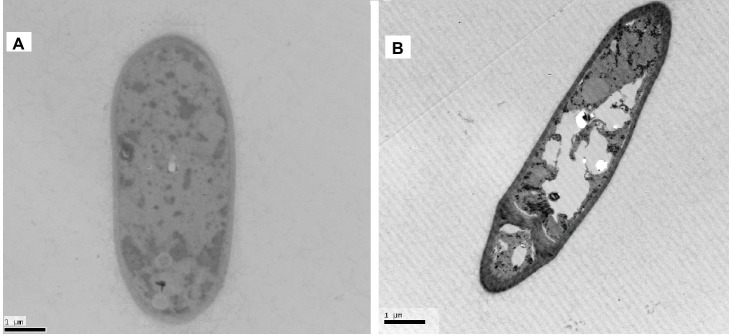
TEM analysis of the hypha of *A. pullulans* interacted with Px-Mor. **(A)** Naive *A. pullulans* hypha; **(B)** the hypha of *A. pullulans* interacted with Px-Mor. The TEM images revealed that there are holes in the mycelium and the cellular cytoplasmic contents were dissolved and became vague **(B)**. The untreated spore **(A)** had a bright and normal smooth surface and the cellular cytoplasmic content was clear.

The effect of *Px-Mor* on the morphology of *A. pullulans* was determined using SEM. The treatment of *A. pullulans* mycelia with moricins at 30 μM for 3 h caused differences in the morphology. In the treatment with moricin, mycelia were short. Spores were wrinkled and showed blebbing as compared to the control (Figure 6). The morphological changes of *A. pullulans* spore after treatment with 30 μM Px-Mor was also examined using TEM. The graph shows that the cell membrane appeared to be wafery and the cellular cytoplasmic contents were dissolved and unclear (Figure 7). We also observed disruption in the cell membrane of *A. pullulans* spores by Px-Mor.

## Discussion

AMPs are part of the innate immune response and present among all classes of life and show remarkable structural and functional diversity. They possess antimicrobial as well as immunomodulatory properties which make them important candidates for the development of novel therapeutics (Fjell et al., 2012; Mahlapuu et al., 2016). The present study described the purification and cloning of Px-Mor, which can be classified in the lepidopteran moricin family, from *P. xyostella*. So far, moricins have been found only in Lepidopteran insects, and cDNAs encoding moricins have been identified in *B. mori* (Furukawa et al., 1999), *M. sexta* (Zhu et al., 2003), *S. litura* (Oizumi et al., 2005), *G. mellonella* (Brown et al., 2008), *H. armigera* (Wang et al., 2010), and *S. exigua* (Jurat-Fuentes et al., 2015). The numbers of amino acid residue of signal peptide or mature peptide and the molecular mass of Px-Mor were similar to those of the other AMPs under moricin family. The solution structure of moricin was determined to have a long α-helix of about eight turns along nearly the full length of the molecule except for several N-terminal and C-terminal residues (Hemmi et al., 2002; Oizumi et al., 2005; Dai et al., 2008). The N-terminal part of the Px-Mor α-helix (residues 5–22) is an amphipathic section and responsible for the increasement in the permeability of the membrane to kill the bacteria, whereas the C-terminal region (residues 23–36) is a hydrophobic section and plays a critical for the antimicrobial activity of moricin (Hemmi et al., 2002). This structure is similar to that of cecropins, except lacking a hinge region in moricins (Yi et al., 2014). Hence, Px-Mor can be classified as a cecropin-type antibacterial peptide, which may play a similar role in the effective defense system of insects against invading pathogens. Furthermore, Px-Mor that derives from transcriptome displayed high identities with three moricins deduced from the genome (You et al., 2013). Phylogenetic analysis indicated that Px-Mor have a high similarity to the other moricins identified from the Lepidopteran insects and have a strong evolutionary relationship with the moricin peptides from *G. mellonella*, *B. mori*, and *D. plexippus*, suggesting they might have derived from a common ancestral gene.

It was shown that silencing of the AMP *gloverin* reduces hatching rate in *B. mori* (Mrinal and Nagaraju, 2008). Similarly, RNAi in *S. exigua gloverin* gene decreases pupation and prolongs larval period (Hwang and Kim, 2011). In this study, Px-Mor mRNA was detected in different developmental stages of diamondback moth and in various tissues of the fourth instar larvae of *P. xyostella*. Px-Mor was highly expressed in fourth instar and prepupa stage, which differentially expressed comparing to other stages. These results suggest that, in addition to have antimicrobial activity, AMP Px-Mor also played a major role in the development. Px-Mor was strongly induced by *B. thuringiensis*, and the expression of Px-Mor reached a peak at 42 h post-infection in the fourth instar larvae, which illustrated that Px-Mor involved in the process of metamorphosis and immune response. In this study, moricin gene expression was observed in the Malpighian tubule as well as in midgut, epidermis, fat body and hemocytes. Px-Mor was mainly expressed in fat body and hemocyte. After induction by *B. thuringiensis*, the expression of Px-Mor reached a high level in the fat body at an earlier stage than in hemocyte. This supported the hypothesis that most of immune genes encoding antibacterial or antifungal peptides are induced by microbial infection, and synthesized predominantly in fat body and then subsequently released into hemolymph (Kanost et al., 2004).

Defense against Gram-positive bacteria and natural fungal infection is mediated by the Toll signaling pathway, whereas defense against Gram-negative bacterial infections is associated with the Imd pathway. It has been reported that expression of moricin can be regulated by the Toll pathway, nuclear factor-κB (NF-κB)/Rel, and GATA transcription factors (Zhong et al., 2012). Cheng et al. (2006) identified NF-κB and GATA binding sites in the promoter regions of the moricin (Cheng et al., 2006). A 22-bp NF-κB-GATA cis-element associated with the enhanced activity of the AMP in *D. melanogaster* and *M. sexta* was also reported in the previous study (Rao et al., 2011). In the present study, the expression levels of Px-Mor in hemolymph were rapidly increased by the injection of heat-inactivated *B. thuringiensis* and *A. pullulans*. In the other species, the expression of HaMor (moricin from *H. armigera*) was increased after fungal infection (Wang et al., 2010). This implied that infection with *B. thuringiensis and A. pullulans* possibly activates the Toll pathway, which mainly responds to Gram-positive bacteria or fungi and the high expression levels of Toll and IMD pathways genes in pupae might be associated with high expression of AMPs (Xia et al., 2015). This is not surprising as early study showed that the recombinant moricin expressed by *E. coli* has consistent activity with the native peptide (Hara and Yamakawa, 1996). In our earlier reports we cloned and expressed both native (Mdcec) and recombinant (Mdcec/6His with native N-terminus) *Musca domestica* cecropin A in *P. patoris*. The study found that both Mdcec and Mdcec/6His possess similar antimicrobial property against bacteria. While Mdcec/6His possess higher activity against fungal infection when compared with the native peptide and the results indicated that the 6His-tag has no negative effect of the protein activity (Jin et al., 2006). It was shown that moricin from *B. mori* have a relatively higher activity against Gram-positive bacteria than cecropin B from *Hyalophora cecropia* which displayed a relatively higher activities against Gram-negative bacteria (Hu et al., 2013). In the present study, to facilitate purification of Px-Mor, the C-terminal recombinant protein Px-Mor was also fused to His-tag and expressed in Drosophila S2 cells. Antimicrobial assays demonstrated that both native and recombinant Px-Mor had high activity against target microbes, and showed that the His-tag fused to the C-terminus had a positive effect on the activity. Similar results were found in previous study where His-tag containing recombinant protein showed high antimicrobial activity (Lundström et al., 2002). It was also reported that strategically placed histidine allows AMP with pH-dependent antimicrobial activity (Lee et al., 1997). Addition of His-tag may increase the affinity of the recombinant proteins toward the bacterial membranes due to the increase in positive charges in C-terminal position (Tavares et al., 2012).

The *widespread use of antifungal agent* during the disease treatment often leads to the development of resistance among the fungal and other microbes which is a major concern mainly for the immunocompromised patients. Thus, the development of novel, safe and efficient antifungal agents is highly needed to combat with the increasing number of resistant fungal isolates (Pfaller et al., 2012; Sanglard, 2016; Seneviratne and Rosa, 2016; Bondaryk et al., 2017). A wide range of new insect AMPs were reported in the last few years mainly due to the availability of the complete genome and transcriptomic data from diverse insect species and showed potentials against various microbial infections. These AMPs are considered as a potential source of alternative antibiotics to fight against antibiotic resistance (Vilcinskas, 2013; Mylonakis et al., 2016). The reported native and recombinant Px-Mor in this study showed high activity against the treated microbial pathogens and can be further exploited for the development of new antifungal drugs against the opportunistic fungi *A. pullulans.*

In conclusion, this study described the characteristics of newly identified AMP Px-Mor from *P. xyostella* and evaluates its activity against Gram-positive and Gram-negative bacterial and fungal infection. Px-Mor was synthesized primarily in fat body and then released into hemolymph. Its expression was highest in fat body and hemocyte. The recombinant protein and synthesized Px-Mor analogues had high activity against the opportunistic fungi *A. pullulans*. Our work elucidated the role of Px-Mor role in the immune responses of *P. xylostella* and supported its potential as a topical antimicrobial agent against pathogens including *A. pullulans.*

## Data Availability Statement

The sequence for Px-Mor has been deposited in GenBank under the accession number KF960047.

## Author Contributions

XX, AZ, YW, BL, PL, JY, SDM, and FJ performed the experiment. XZ, WJ, and SDM analyzed the data. XX, FJ, and SDM reviewed data, performed statistical analysis, planned and coordinated the study, and wrote the manuscript. All authors revised and approved the final version of the manuscript.

### Conflict of Interest

The authors declare that the research was conducted in the absence of any commercial or financial relationships that could be construed as a potential conflict of interest.

## Footnotes

^1^http://www.ncbi.nlm.nih.gov/

^2^http://www.expasy.ch/SingalP

^3^http://www.expasy.org/tools/pi-tool.html

^4^http://www.scripps.edu/~cdputnam/protcalc.html

^5^http://www.ebi.ac.uk/tools/clustalw2

^6^http://www.sbg.bio.ic.ac.uk/phyre2/html/page.cgi?id=index

## Supplementary Material

SUPPLEMENTARY FIGURE S1 Predicted structures of Px-Mor, using Phyre 2. **(A)** Secondary structure. **(B)** Tertiary structure.

SUPPLEMENTARY TABLE S1 Structures and characteristics of the Px-Mor analogues.
