# Supplementary figures and images for: Molecular Identification of a Moricin Family Antimicrobial Peptide (Px-Mor) From Plutella xylostella With Activities Against the Opportunistic Human Pathogen Aureobasidium pullulans
Source: Front Microbiol. 2019 Oct 11;10:2211. doi: 10.3389/fmicb.2019.02211 (PMC6797621; doi:10.3389/fmicb.2019.02211)

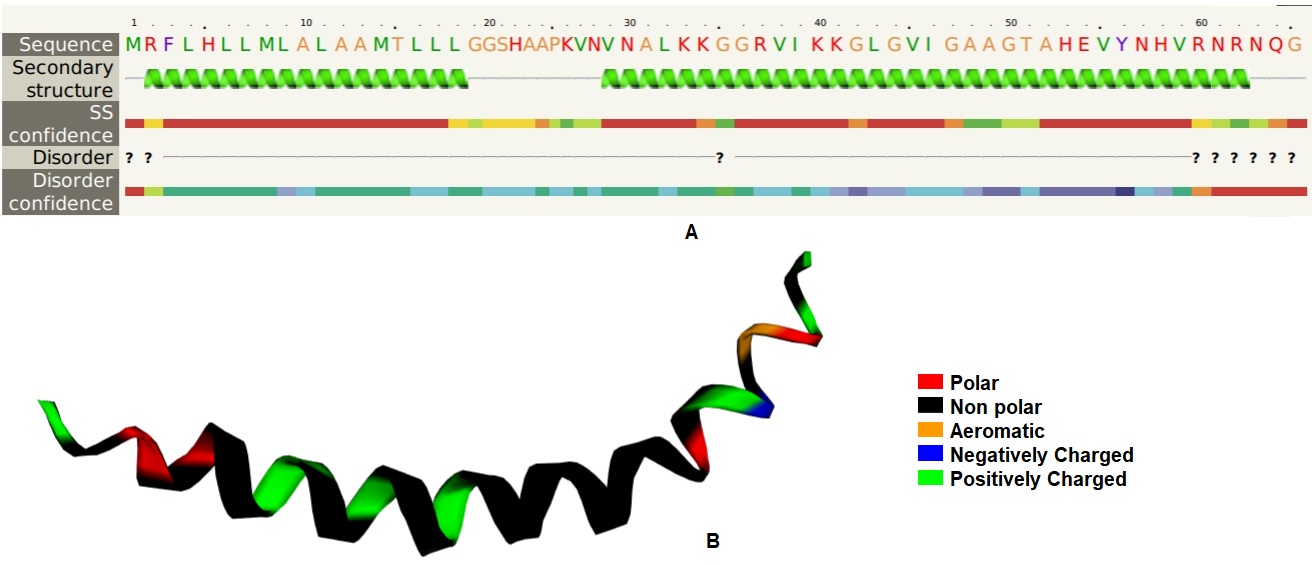

Supplement: SUPPLEMENTARY FIGURE S1 — Predicted structures of Px-Mor, using Phyre 2. (A) Secondary structure. (B) Tertiary structure. [file Image_1.JPEG]
