# Supplementary material for: Molecular Identification of a Moricin Family Antimicrobial Peptide (Px-Mor) From Plutella xylostella With Activities Against the Opportunistic Human Pathogen Aureobasidium pullulans
Source: Front Microbiol. 2019 Oct 11;10:2211. doi: 10.3389/fmicb.2019.02211 (PMC6797621; doi:10.3389/fmicb.2019.02211)
Supplement: SUPPLEMENTARY TABLE S1 — Structures and characteristics of the Px-Mor analogues. [file Table_1.docx]

Supplementary Table 1**.** Structures and characteristics of the Px-Mor analogues

|  | **Name** | **Accession No** | **Structure** | **Theoretical pI** | Charge at pH 7.00 | **Mw (average)** | **Species** |
| --- | --- | --- | --- | --- | --- | --- | --- |
| 1 | Px-moricin-recombinant | KF960047 | 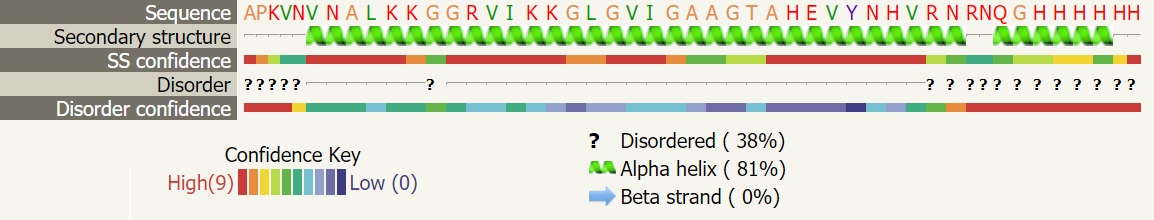 | 11.17 | 8.8 | 5215.93 | *P. xylostella* |
| 2 | Px010113 | Px010113 | 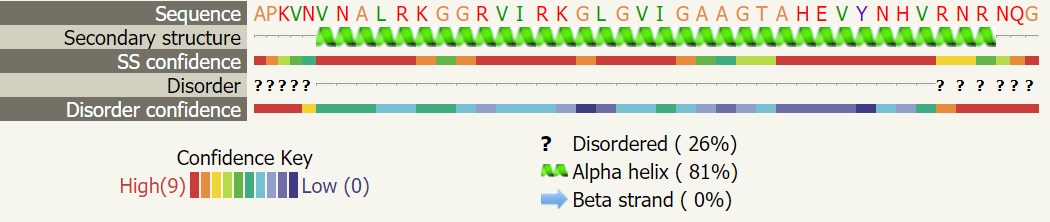 | 11.84 | 7.4 | 4449.11 | *P. xylostella* |
| 3 | Px010115 | Px010115 | 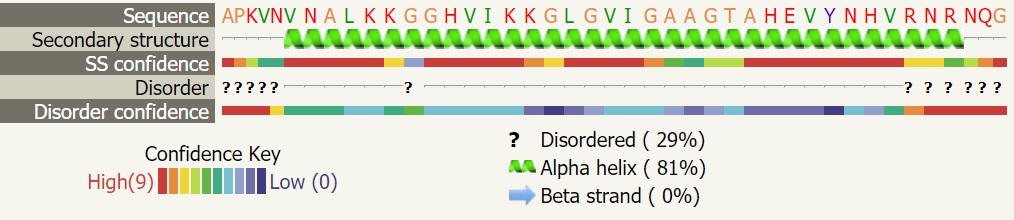 | 10.66 | 6.6 | 4374.04 | *P. xylostella* |
| 4 | Pxmor3 | KX164502 | 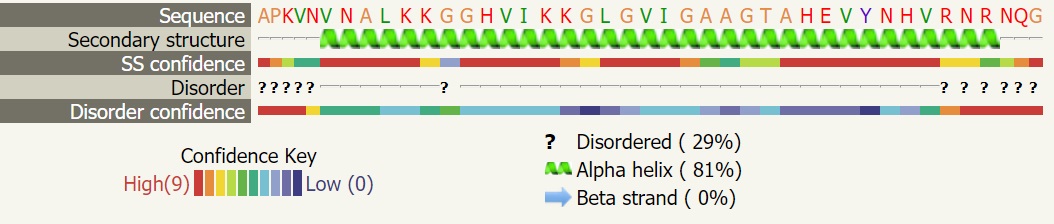 | 10.66 | 6.6 | 4374.04 | *P. xylostella* |
| 5 | Pxmor1/Pxmor2 | KX164500/KX164501 | 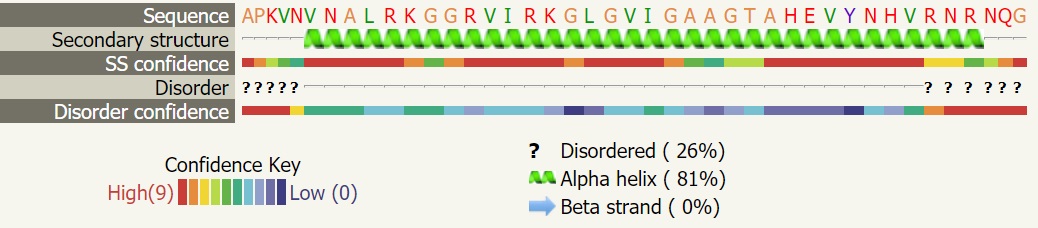 | 11.84 | 7.4 | 4449.11 | *P. xylostella* |
| 6 | Ms-moricin | AY232301 | 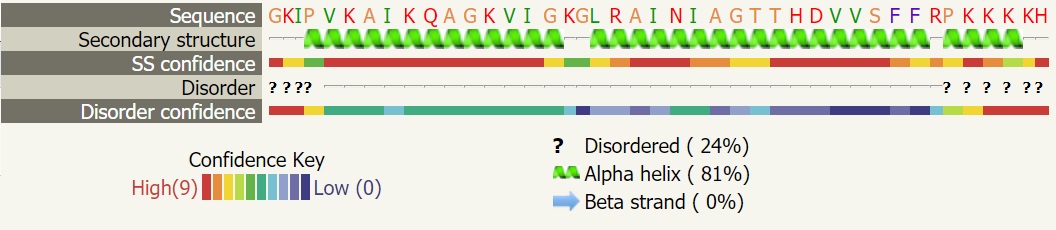 | 11.36 | 10.4 | 4538.50 | *Manduca sexta* |
| 7 | Se-moricin | AY611631 | 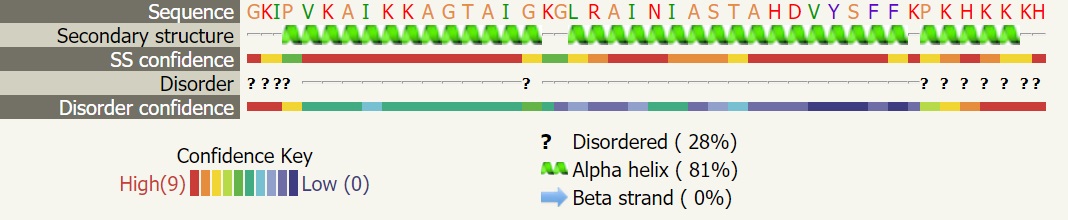 | 10.64 | 10.6 | 4656.59 | *Spodoptera exigua* |
| 8 | Sl-moricin | AB100428 | 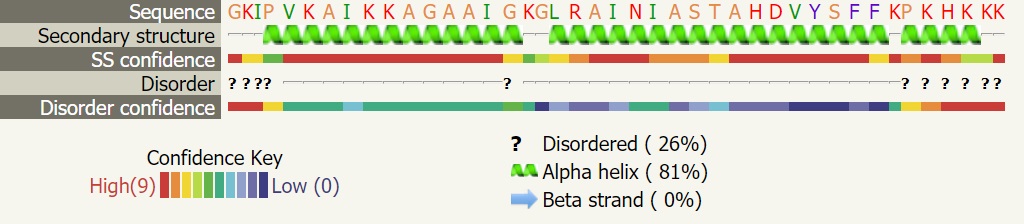 | 10.64 | 10.4 | 4489.42 | *Spodoptera litura* |
| 9 | Hv-moricin | P83416 | 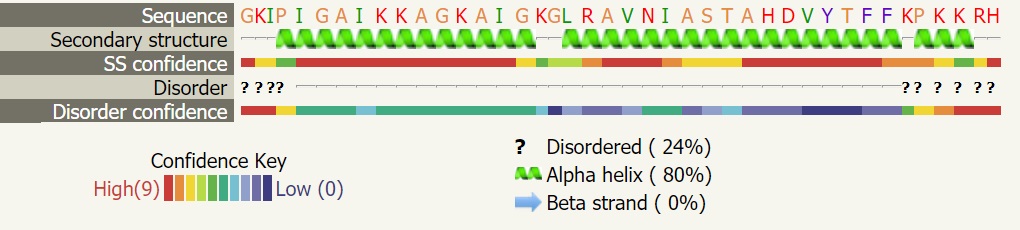 | 10.84 | 9.4 | 4389.26 | *Heliothis virescens* |
| 10 | Ap-moricin | DQ519400 | 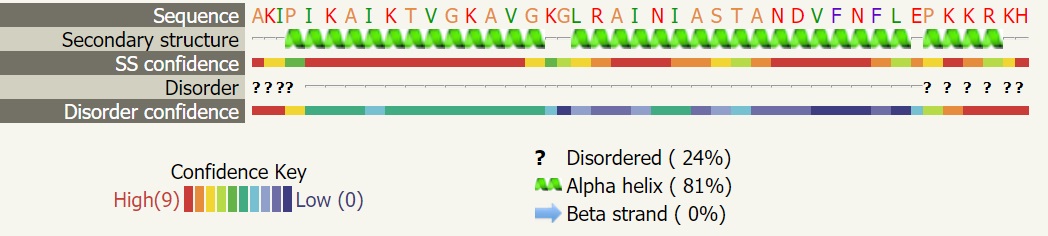 | 10.79 | 8.1 | 4544.46 | *Antheraea pernyi* |
| 11 | Hp-moricin | AY847955 | 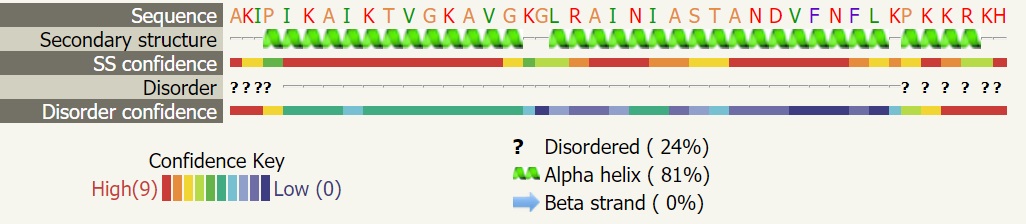 | 11.36 | 10.1 | 4543.52 | *Hyblaea purea* |
| 12 | Lo-moricin | CX816233 | 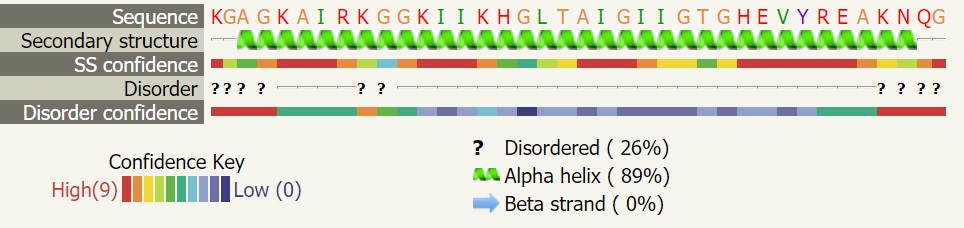 | 10.38 | 6.4 | 3928.60 | *Lonomia obliqua* |
| 13 | Gm-A-moricin | EF564365 | 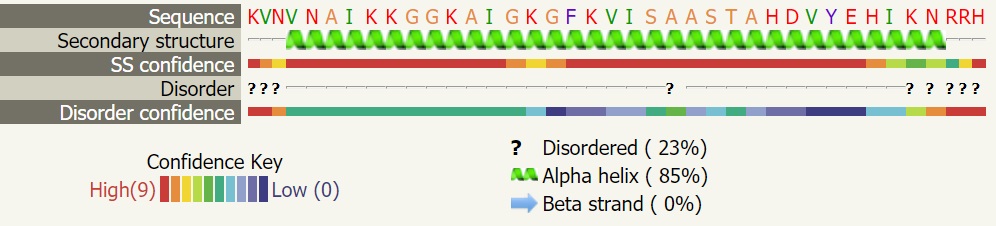 | 10.46 | 7.6 | 4243.93 | *Galleria mellonella* |
| 14 | Gm-B-moricin | EF564366 | 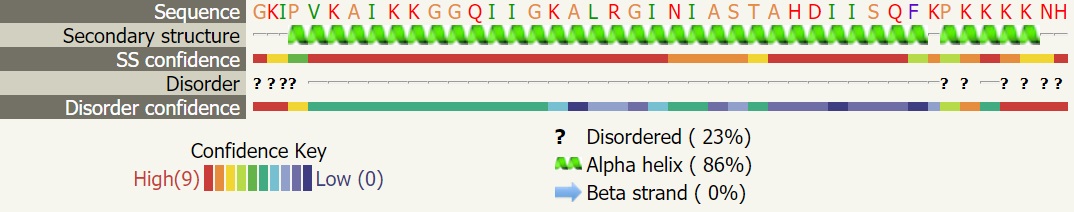 | 10.92 | 10.4 | 4633.60 | *Galleria mellonella* |
| 15 | Gm-D-moricin | EF564372 | 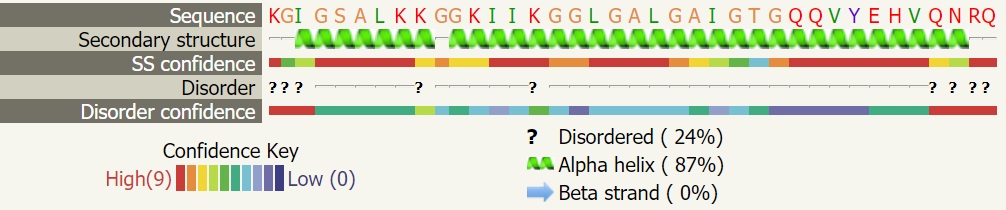 | 10.30 | 5.1 | 3833.45 | *Galleria mellonella* |
| 16 | Gm-C1-moricin | EF564367 | 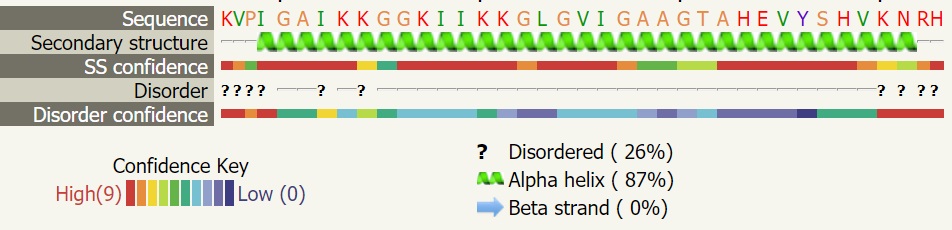 | 10.47 | 7.6 | 3933.71 | *Galleria mellonella* |
| 17 | Gm-C2-moricin | EF564368 | 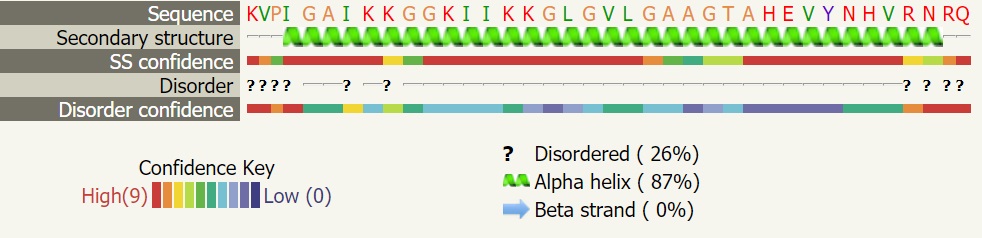 | 10.73 | 7.4 | 3979.73 | *Galleria mellonella* |
| 18 | Gm-C3-moricin | EF564369 | 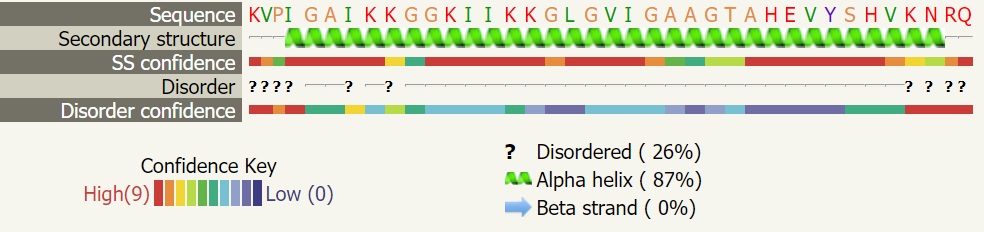 | 10.47 | 7.4 | 3924.69 | *Galleria mellonella* |
| 19 | Gm-C4-moricin | EF564370 | 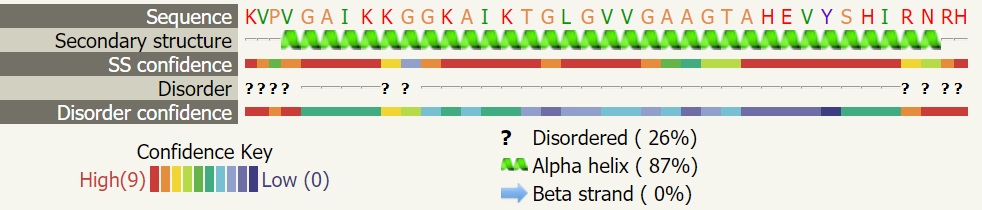 | 10.66 | 6.6 | 3878.54 | *Galleria mellonella* |
| 20 | Gm-C5-moricin | EF564371 | 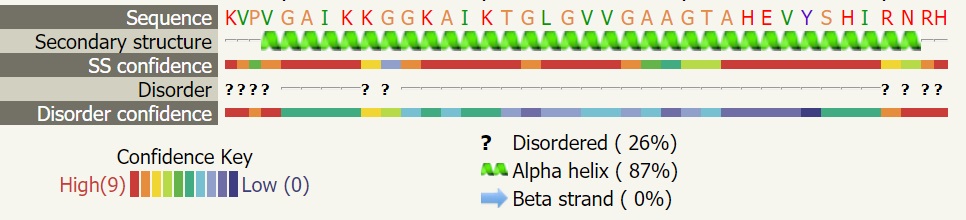 | 10.66 | 6.6 | 3878.54 | *Galleria mellonella* |
| 21 | BmA1-moricin | AB006915 | 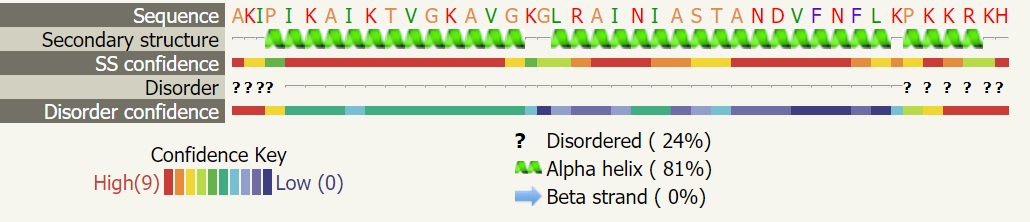 | 11.36 | 10.1 | 4543.52 | *Bombyx mori* |
| 22 | BmB1-moricin | CH380045 | 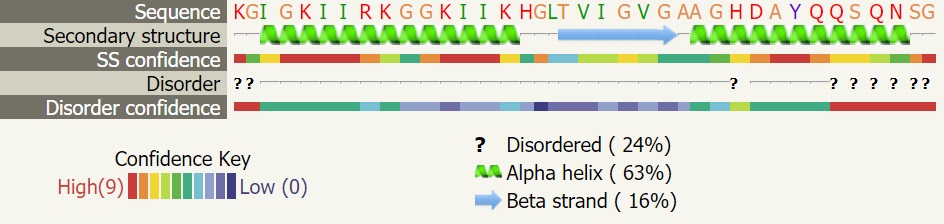 | 10.30 | 5.4 | 3858.46 | *Bombyx mori* |
| 23 | BmB2-moricin | CH380045 | 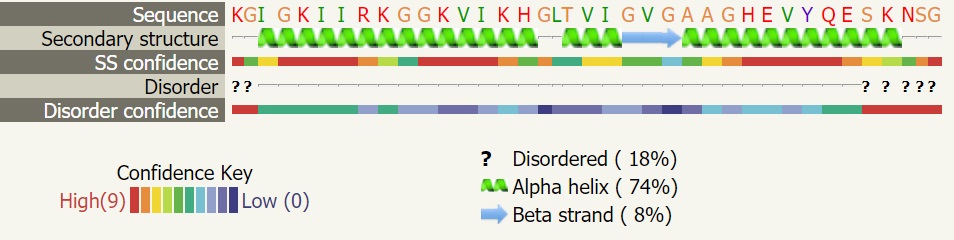 | 10.12 | 5.4 | 3887.54 | *Bombyx mori* |
| 24 | BmB3-moricin | CH380569 | 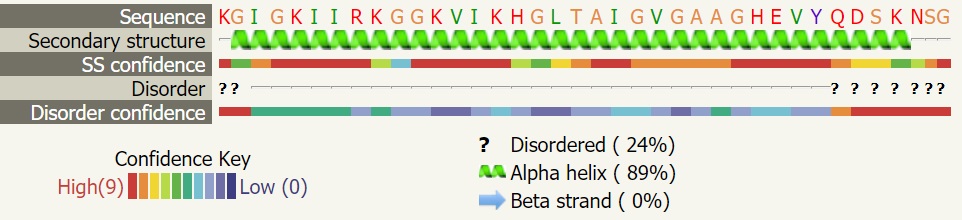 | 10.12 | 5.4 | 3845.46 | *Bombyx mori* |
| 25 | BmB6-moricin | CH380569 | 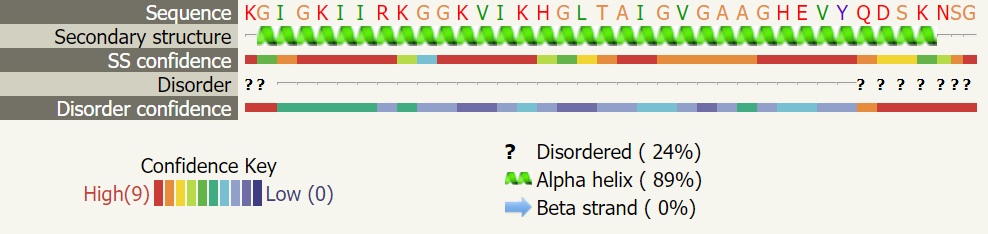 | 10.12 | 5.4 | 3845.46 | *Bombyx mori* |
| 26 | BmB8-moricin | CH380569 | 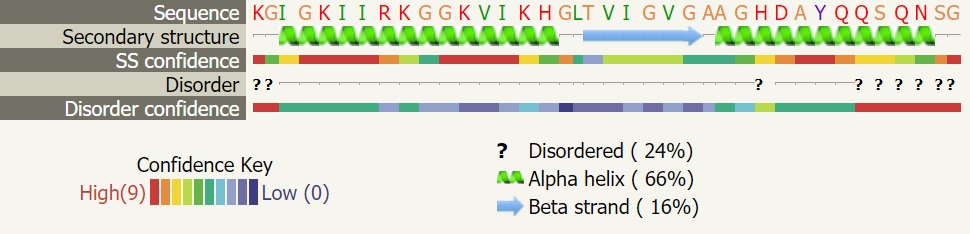 | 10.30 | 5.4 | 3844.44 | *Bombyx mori* |
| 27 | Bm-A2-moricin | CH391671 | 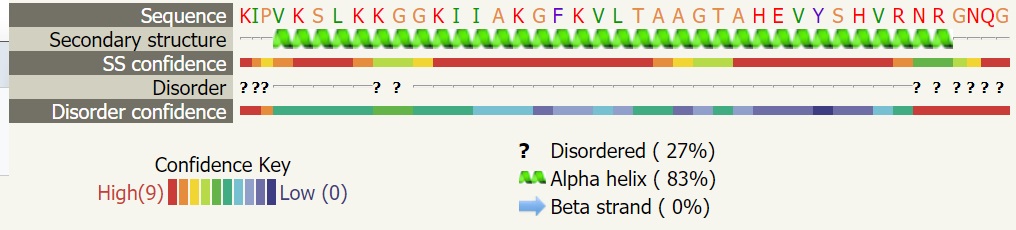 | 10.79 | 8.4 | 4360.13 | *Bombyx mori* |
| 28 | Bm-A3-moricin | AADK01025872 | 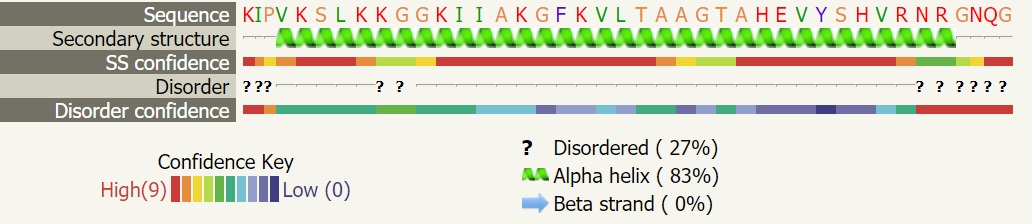 | 10.79 | 8.4 | 4360.13 | *Bombyx mori* |
| 29 | Bm-A4-moricin | AV402493 | 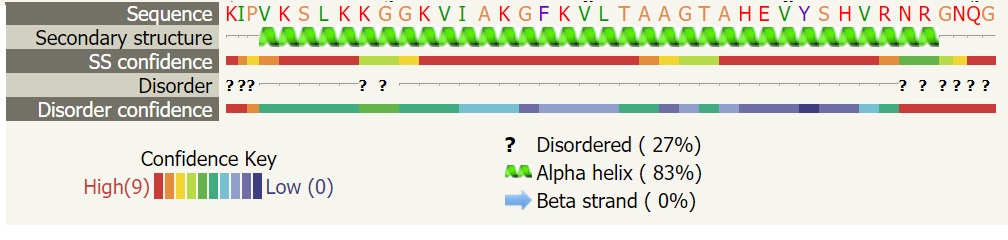 | 10.79 | 8.4 | 4346.11 | *Bombyx mori* |
| 30 | Ci-P1647-moricin | WO2004/016650 | 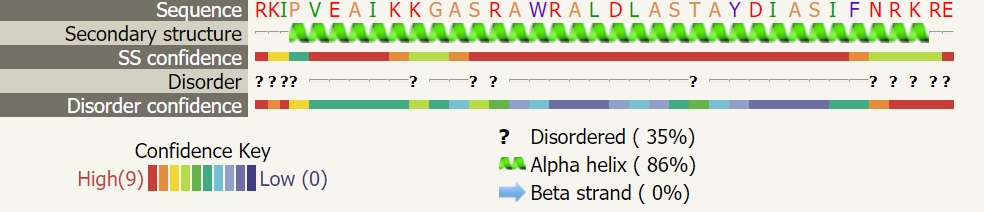 | 10.52 | 4.9 | 4202.88 | *Caligoillioneus* |
| 31 | Ci-P1648-moricin | WO2004/016650 | 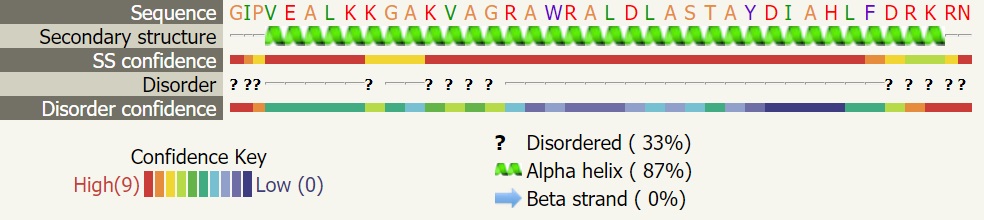 | 10.16 | 4.2 | 4279.96 | *Caligoillioneus* |
| 32 | Ci-P1646-moricin | WO2004/016650 | 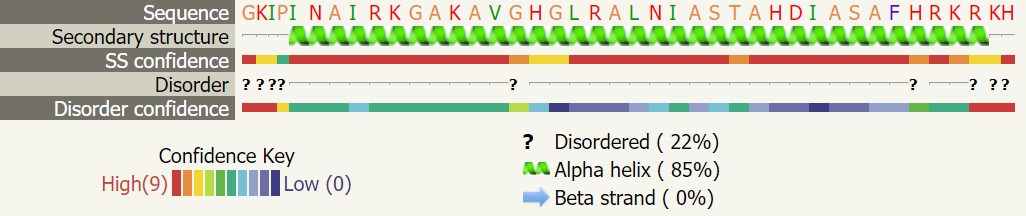 | 12.02 | 8.9 | 4383.14 | *Caligoillioneus* |

-
